# Bioinformatics Analysis and Validation of the Expressed Sequences Tag in Human Colorectal Adenocarcinoma

**DOI:** 10.4021/gr2009.04.1287

**Published:** 2009-03-20

**Authors:** Yao Chen, Chun Le Zhang, Yong Qiang Shen, Li Cheng Wang

**Affiliations:** a Department of Anatomy, Basic Medical and Legal Medical Institute of Sichuan university. Chengdu, Sichuan, China. 610041

**Keywords:** Bioinformatic analyses, Expressed sequence tag, Colorectal adenocarcinoma

## Abstract

**Background:**

This study was to investigate some new pathological genes in colorectal adenocarcinoma of human.

**Methods:**

Human colorectal adenocarcinoma tissues and normal colorectal tissues were taken and suppression subtractive hybridization (SSH) and cDNA microarray techniques were employed. From differentially expressed 86 expressed sequence tags (EST), 10 EST of the SSH were selected as seed sequence for bioinformatics analyses, semi-quantitative RT-PCR and PCR-sequencing. Each lane of semi-quantitative RT-PCR was analyzed by Q1 software.

**Results:**

Among these 10 EST, it has been found that ES274070, ES274071, ES274076 and ES274081 may play role in the onset of colorectal adenocarcinoma in human.

**Conclusions:**

The ES274070, ES274071, ES274076 and ES274081 are related to the onset of human colorectal adenocarcinoma.

## Introduction

The initiation and development of colorectal adenocarcinoma are multistep process that is involved multi-gene changes. During this process, cell division and differentiation are abnormally regulated by the many genes [1]. It will be benefit to the early diagnosis, effective treatment and prevention if molecular pathological mechanism of human colorectal adenocarcinoma are elucidated. With the accomplishment of human genome program, genome research has entered into a new phase of gene abstraction and data analysis. Cloning novel genes by means of bioinformatics has become a new strategy [2].

This study aims to screen and clone the genes related to genesis of human colorectal carcinoma and further study their function and relationship with the colorectal adenocarcinoma. We selected 10 expressed sequence tags (EST) from suppression subtractive hybridization (SSH) combining cDNA microarray as seed sequences to extend them in order to acquire full-length cDNA and validate expressiong by RT-PCR. It will be helpful for us to further study their function; moreover, this will make us find early diagnostic and curative targets.

## Materials and Methods

The normal human colorectal tissues and the human colorectal adenocarcinoma (HCRAC) tissue samples from HCRAC patients in West China Hospital of Sichuan University were snap-frozen in liquid nitrogen immediately after surgery and stored at -80°C. Nine pairs of HCRAC and normal colorectal tissues had been used, isolation of RNA was performed by the TRIzol method (Invitrogen) according to manufacturer’s instructions. The SSH combined with cDNA microarray was performed [3]. The 10 EST that include ES274070, ES274071, ES274073, ES274075, ES274076, ES274077, ES274081, ES274083, ES274084 and ES274085 were selected sequences for further bioinformatics analysis. The first step of bioinformatics analysis is to choose the EST as seed sequence and to find its matching sequence. The second step is to extend those EST as long as possible through the extension method of matching sequences blast cycling. That is to assemble these matching sequences together to form a longer EST and then use the new EST to conduct blast retrieval to find more matching sequences. This process is repeated until no more matching sequences can be found. Thus, a contig is obtained. Then we analyzed the obtained full-length cDNA sequence by bioinformatics softwares and databases on network. Semi-quantitative RT-PCR and PCR-sequencing were done (Table 1, 2). Each lane of semi-quantitative RT-PCR was analyzed by Q1 software. Then t test was done using SPSS13.0 statistic software.

**Table 1:** PCR conditions

Accession number	PCR condition
ES274070	94°C 3 min; 94 °C 30 sec, 54 °C 30 sec, 72 °C 1 min, 30 cycles; 72 °C 5min
ES274071	94 °C 4 min; 94 °C 30 sec, 52.5 °C 30 sec, 72 °C 2 min, 30 cycles;72 °C 7min
ES274076	94 °C 4 min; 94 °C 30 sec, 52 °C 30 sec, 72 °C 2 min, 33 cycles; 72 °C 7min
ES274073	94 °C 4 min; 94 °C 30 sec, 57 °C 30 sec, 72 °C 2 min, 33 cycles; 72 °C 7min

**Table 2:** PCR primers and Products

Accession number	Primer	Product
ES274070	Forward 5’ –TTGGAGCCCTGAGTATCTGTG -3’ Reverse 5’ –TAATGGAACCTGGTGCTAAGTC -3’	616bp
ES274071	Forward 5’-CCTTCGCCTTCCCTTCTC-3’ Reverse 5’-CGACTGAGCACAAGAGGGA-3’	700bp
ES274076	Forward 5’ –GCACTGCCAAGATAGACAA -3’ Reverse 5’ –CTGGAACCTGCTACGAAT -3’	512bp
ES274073	Forward 5’ –TACCACCTACCTCCCTCA -3’ Reverse 5’ –ATGGGCTGGGTTTTACTA -3’	791bp

## Results

**Figure 1:**
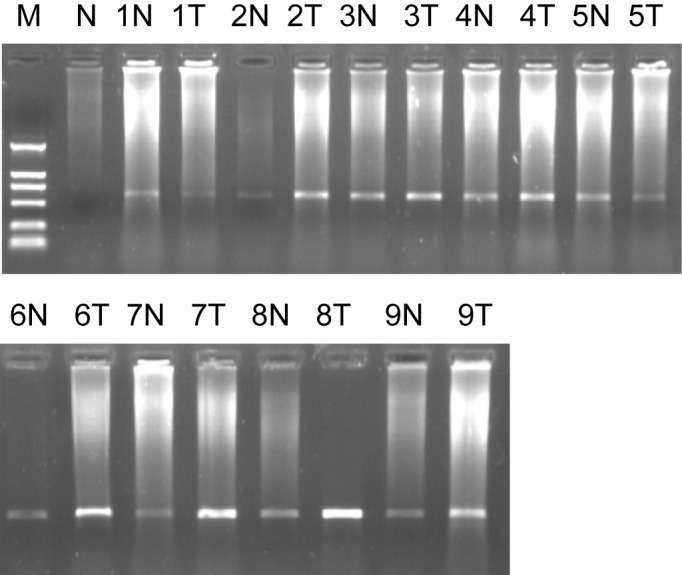
Marker: DL2000, 1% agarose ORF of ES274070 semi-quantitative RT-PCR. 1, 2……9: sample number; N: normal colorectal tissues; T: colorectal adenocarcinoma tissues.

We obtained 1260 differentially expressed clones by SSH. The cDNA microarray showed total 86 EST being identified were more than half past one fold or two-fold differentially expressed genes between two tissues. After they had been sequenced and analyzed by bioinformatics analysis, the sequence data were accepted by Genebank and were identified as EST and got genebank accession number and dbEST number. The 1721bp length cDNA sequence for ES274070 was formed by DNAStar Software and it located in human chromosome 17q21.2. The open reading frame (ORF) was predicted and its product contains 111 amino acids, its molecular weight is 12.8KD, PI is 8.79. On NCBI website, it is found that it has the homology 1649bp sequence (BC042012). The Serial analysis of gene expression (SAGE) indicates that there is expression in oligodendroglioma, astrocytoma, prostatic carcinoma, ovarian cancer, breast cancer, and carcinoma of testis, sarcoenchondroma, and high expression in brain tumor tissue. Semi-quantitative RT-PCR results for its’ ORF were showed that its mRNA in colorectal cancer tissue was significantly higher than that of normal colorectal tissues (P = 0.011), (Fig. 1).

**Figure 2:**
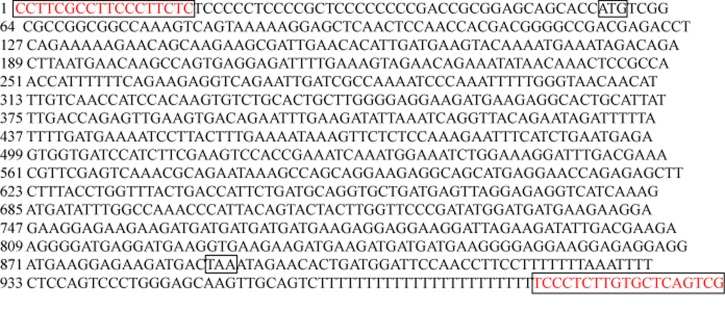
The result of ES274071 ORF sequencing.

The 2790bp length cDNA sequence for ES274071 was also formed. The sequence extends 1550bp from 5’end and 820bp from 3’end. It is located in human chromosome 9q34, ORF’s length is 834bp. Its coded protein is SET translocation, we found the registered sequence NM003011 which is homologous with the assembled sequence, the length is 2577bp. We assembled the full-length cDNA by extending 309bp compared with NM003011 by RT-PCR. The result of ES274071 ORF was shown by sequencing and bioinformatics analysis, it was extended 16bp compared with NM003011 and we got accession number EF534308 (Fig. 2).

**Figure 3:**
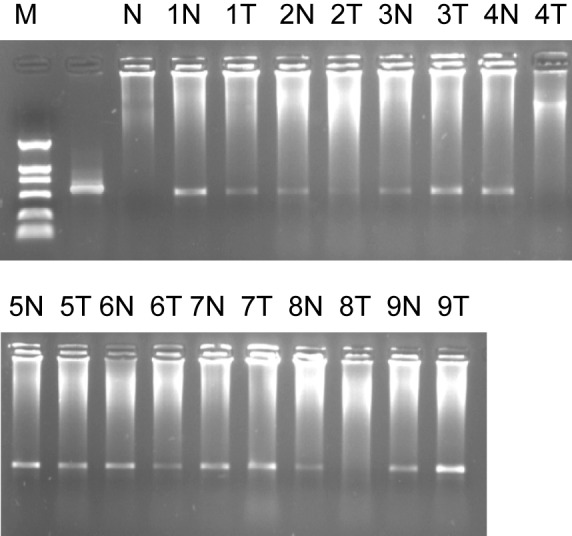
Marker: DL2000, 1% agarose ORF of ES274076 semi-quantitative RT-PCR. 1, 2……9: sample number; N: normal colorectal tissues; T: colorectal adenocarcinoma tissues.

We also found that ES274073 corresponding full-length sequence were human mitochondrial genome (NM183047). Semi-quantitative RT-PCR results of NM183047 showed there was no significant difference in normal tissues and colorectal cancer tissues (P > 0.05). The ES274076 corresponding full-length sequence was human zinc finger protein (NM002644), semi-quantitative RT-PCR results of its ORF showed that its ORF in colorectal cancer tissue was lower than that of normal organizations (P = 0.044), (Fig. 3).

In fact we found that two EST (ES274075 and ES274084) are the same sequence. Its coded protein may be human collagen protein type. ES274077 located in human chromosome 3q24.3. Using the software DNAStar to assemble the matching sequences together to form a 4503bp cDNA sequence. The ORF was predicted and its product contains 268 amino acids. Its product is ELL associated factor-1 (EAF-1). The SAGE indicates that there is expression in oligodendroglioma, astrocytoma, prostatic carcinoma, ovarian cancer, breast cancer, carcinoma of testis, sarcoenchondroma, and high expression in brain tumor tissue. ES274081 located in human chromosome 20q13.1. The ORF was predicted and its product contains 432 amino acids. Its product is hypothetical protein LOC388969. The SAGE indicates that there is expression in cervical tumor, gastrointestinal tumor, glioma, head and neck tumor, leukemia, lung tumor, ovarian tumor, pancreatic tumor, and high expression in head and neck tumor tissue. ES274083 is located in human chromosome 2q11.2. The ORF was predicted and its product contains 268 amino acids. Its product is S-adenosylhomocysteine hydrolase (SAHH). The SAGE indicates that there is expression in breast (mammary gland) tumor, cervical tumor, colorectal tumor, leukemia, liver tumor, lung tumor, lymphoma, retinoblastoma, and high expression in retinoblastoma tissue. ES274085 is located in 13 chrosome (103-313bp) and 14 chrosome (1-103bp).

## Discussion

The ORF of ES274070 located in BC042012 code the kind of hypothetical protein LOC90110, until now, there is its function report [4]. In our study, semi-quantitative RT-PCR results showed that ES274070 in colorectal cancer tissues was up-regulated; so, further study is needed to identify the its relationship with human colorectal adenocarcinoma. The coded protein of ES274071 was found to be SET translocation. SET is a suppressor of PP2A and NM23. PP2A is a kind of phosphorylase and is a tumor suppressor protein, its over-expression could lead to disorder of cell cycle, and it contributes to cell proliferation, growth and division. It plays some role in the cell apoptosis mediated by caspases. SET has a non-dependent nuclesome assembly protein; its NAP activity can enhance the affinity of chromatin [5, 6]. ES274073 located in the 8590-9000 of human mitochondria, its’ ORF of gene is mitochondrially encoded ATP synthase 6. Some authors reported that the mutation and down-regulated expressed mitochondrially encoded ATP synthase 6 is related to heart disease and disorder of neural system [7-9]. In our study, ES274073 was not significantly different between normal colorectal tissues and colorectal cancer tissues (P > 0.05). The protein encoded by ES274076’s gene is a receptor for activated C-kinase (RACK) protein. The encoded protein has been shown to bind in vitro to activated protein kinase C beta I. In addition, this protein is a cutaneous T-cell lymphoma-associated antigen. Finally, the protein contains a bromodomain and two zinc fingers, and is thought to be a transcriptional regulator. Multiple transcript variants encoding several different isoforms have been found for this gene [10-12]. Semi-quantitative RT-PCR results showed that ES274076 in colorectal cancer tissues was down-regulated; probably, activated protein kinase C beta I (PKCβI)will be reduced, so the sensitivity of cancer cells for anti-tumor factor (TNFα and others) will decrease and promote the growth of cancer cells and inhibit apoptosis of cancer cells.

ES274077encoded protein is ELL associated factor-1 (EAF-1), which is a positive regulation protein for ELL and RNA polymerase II. EAF-1 has been found to have a dominant effect in leukemogenesis [13-15]. The function of the hypothetical protein LOC388969 for ES274081 is unclear now. But it is shown that it expresses in various types of human tumors, this knowledge is helpful to guide our further study. The protein production for ES274083 is S-adenosylhomocysteine hydrolase (SAHH). Although SAGE shows that there is expression in many human tumor tissues, it has not been found that there is relationship between colorectal adenocarcinoma (CRA) and SAHH [16, 17]. The other two EST, ES274084 and ES274075, after analysis, we found that they are the same sequence, its coded protein may be human collagen protein type I [18].

In conclusion, with the development of molecular biology, more and more data need to be analyzed, this is possible because of the development of computer and the other sciences and techniques, so the bioinformatics have became very useful tool and new approaches. The colorectal adenocarcinoma is a common malignant and a multi-genes and multi-steps disease. Through the bioinformatics analyses, we are able to screen the data from cDNA microarray, thus we avoid wasting of time and manpower. It is very helpful for us to search the pathologic genes associated with the development of human colorectal adenocarcinoma. Furthermore, ES274070, ES274071 and ES274076 and ES274081 were shown related to onset of human colorectal adenocarcinoma.
